# Interventions to reduce adverse health outcomes resulting from manifestations of gender bias amongst immigrant populations: a scoping review

**DOI:** 10.1186/s12905-018-0604-2

**Published:** 2018-06-19

**Authors:** Alia Januwalla, Ariel Pulver, Susitha Wanigaratne, Patricia O’Campo, Marcelo L. Urquia

**Affiliations:** 1 Centre for Urban Health Solutions, Li Ka Shing Knowledge Institute, St. Michael’s Hospital, 209 Victoria Street, Toronto, ON M5B 1W8 Canada; 2 Dalla Lana School of Public Health, University of Toronto, 155 College Street, 6th floor, Toronto, ON M5T 3M7 Canada; 3 Rady Faculty of Health Sciences, S113 Medical Services Building – 70 Bannatyne Avenue, University of Manitoba, Winnipeg, MB R3E 0W3 Canada

**Keywords:** Immigrant health, Gender bias, Gender-based violence, Interventions, Violence against women

## Abstract

**Background:**

Immigrants to Western countries increasingly originate from countries with pervasive gender inequalities, where women experience disproportionately high rates of threats to their well-being. Health and social services in countries of settlement encounter several adverse outcomes linked to gender bias among immigrant groups. Little is known about interventions implemented to address manifestations of gender bias among immigrant populations.

**Methods:**

A scoping review was undertaken to describe the literature on existing interventions and determine knowledge gaps. Nine academic and grey literature databases were searched for literature, with four reviewers screening the results.

**Results:**

Of the 29 included reports, most targeted domestic violence amongst the Latino population in the United States, with few interventions focusing on other outcomes, populations, and settings. The majority reported achieving their objective, although 13 interventions were not evaluated.

**Conclusions:**

Future research and practice to address gender bias among immigrants may benefit from expanding on ethnic diversity, designing and reporting evaluations, addressing the context of gender inequities, tailoring to local community needs, and engaging community-based groups.

**Electronic supplementary material:**

The online version of this article (10.1186/s12905-018-0604-2) contains supplementary material, which is available to authorized users.

## Background

Gender biases rooted in patriarchal gender roles have a negative impact on women and girls’ health and wellbeing and persist globally. The World Development Report estimates that women lose more Discounted Health Years of Life to gender-based violence than to breast cancer, cervical cancer, heart disease, AIDS, motor vehicle accidents or war combined [1]. In addition to sexual and domestic violence, many gender-biased practices include female genital circumcision (FGC), forced child marriage, or sex-selective abortion, which are linked to specific customs and beliefs about the societal position of girls and women [2].

The Gender Inequality Index (GII) measures gender inequality at the country level and is considered a marker of women’s disadvantage [3]. The GII reflects the loss in potential human development due to disparity between men and women in the dimensions of reproductive health, empowerment, and labour. The GII is estimated annually amongst 138 countries, including Western high immigrant-receiving countries such as Canada, the United States, United Kingdom, and Australia, as well as most of their top immigrant source countries [3, 4]. The GII demonstrates that even Western immigrant-receiving countries need to make considerable progress to achieving gender equality - for example, Canada and the United States ranked 25th and 55th, respectively, out of 138 countries in 2015 [3, 5]. Important, however, is the indication that source countries of immigration score much higher on the GII (e.g., India and Pakistan ranked 130th and 121st respectively), suggesting that the social norms constructing patterns of gender inequity may persist in Western countries of settlement.

Migration trends may play a significant role in importing cultural and traditional conventions that influence gender disparities in immigrant communities. Furthermore, the challenges associated with migration such as precarious employment, language barriers, financial insecurity, spatial and social isolation, and even legal status may exacerbate existing gender biases against immigrant women [6]. Because of these unique circumstances, immigrant women may experience barriers such that population-wide prevention and/or treatment strategies are ineffective. The unique manifestations of gender bias amongst immigrants, and the subsequent need for tailored interventions, signifies a specific sub-population of interest, and a significant area of focus for social service providers, health professionals, and researchers working with immigrant populations. However, little is known about what interventions have been implemented to address the negative health effects of gender inequities in immigrant communities, and their effectiveness in countries of settlement.

The purpose of this review are two-fold: i) to summarize the literature on interventions aimed at mitigating adverse health effects of gender inequity among immigrant populations, and ii) to identify knowledge gaps and lessons learned from the interventions with the aim to inform future research and practice. To our knowledge, such a synthesis does not exist. This is of increasing importance primarily because of current global migration trends, and because of the need to provide services for this growing and changing population.

## Methods

Our scoping review methodology was guided by frameworks proposed by Arksey and O’Malley (2005) and Levac, Colquhoun, and O’Brien (2010) [7, 8]. Because the purpose of this review was to map out all relevant literature, as well as identify knowledge gaps, the research objectives and search strategy were intended to be broad enough in order to capture the full extent of the literature, but also specific enough to focus on the health effects of inequitable gender relationships within the immigrant population.

For the purposes of this review, we defined an intervention as “an action or set of actions purposely implemented to change health-related outcomes or modify health behaviours that lead to specific outcomes within a population”. We were interested in examining interventions that were aimed at or involved immigrants as participants. Interventions specifically had an objective of reducing outcomes directly related to the physical and/or mental health and well-being of individuals, but were rooted in gender inequity; outcomes that typically disadvantage *women*’*s* health. Possible outcomes, as indicated by the literature [1], could be but were not limited to, female genital circumcision, sexual violence, domestic violence, ‘honor-violence’, sex-selective abortion, and femicide; some of which often uniquely affect women within immigrant communities. As we were interested in focusing on particular health outcomes, we did not include social or economic outcomes such as health care utilization or employment training programs. These gender-based outcomes were instead chosen because they derive from patriarchal relationships in the countries of origin, and are often perpetrated by immigrants reproducing these relationships after migration [9].

### Search strategy

#### Peer reviewed electronic sources

This review was conducted through systematic searches of electronic library databases, including Medline, Scopus, Web of Science, CINAHL, and Gender Studies. The electronic search included sources published from date of inception to May 2016 across all databases. Restrictions were applied to include only English language articles. The search strategy was developed in collaboration between all authors and with consultation from an information specialist.

Three central components of the research question were used in combination, and guided the search. The search combined focused key-word search terms and Boolean search terms. Keywords were searched using truncation symbols when appropriate to capture comprehensive results. Search terms within each theme were combined with the Boolean Operator OR. Themes were combined using the Boolean Operator AND. Below are the search terms used:

### Immigration

MeSH terms included: “Emigrants and Immigrants”, “Emigration and Immigration”, “Refugees”, and “Transients and Migrants”; keywords included: “immigra*”, “emigra*”, “asylum seeker”, “foreign born”, “refugee*”, “migrant*”, “migration”.

### Intervention

MeSH terms included: “Health Promotion”, “Community Health Services”; keywords included: “health promotion”, “community health service*”, “intervention*”, “program*”, “evaluation*”.

### Gender outcome

MeSH terms included: “Sexism”, “Rape”, “Spouse Abuse”, “Battered Women”, “Violence”, “Domestic Violence”, “Intimate Partner Violence”, Circumcision, “Female”, “Sex Offenses”, “Machismo”, “Infanticide”; keywords included: “gender-based violence”, “gender bias”, “gender discrimination”, “violence against women”, “rape*”, “assault*”, “spous* abuse”, “battered women”, “violence”, “female genital circumcision”, “female genital mutilation”, “female genital cutting”, “sex offenses”, “patriarch*”, “gender inequ*”, “gender disparit*”, “machismo”, “misogyny”, “abortion”, “sex selection”, “sex preference”, “infanticide”, “feticide”, “foeticide”, “femicide”.

#### Grey literature

A variety of grey literature databases were searched to identify any relevant but not peer-reviewed literature, such as theses, dissertations, or reports. These databases were OAIsters, the Networked Digital Library of Theses and Dissertations, Google Scholar, and Google. These databases were chosen based on their international scope and variety in terms of types of literature. In order to obtain a reasonable number of results generated with keyword searches, the grey literature search strategy was more limited than the academic search. Furthermore, initial scanning of results using an expanded search strategy yielded results that were irrelevant to the study question (i.e. language instruction or newcomer employment training). For these reasons, the terms “immigrant*” and “intervention*” had to both be in the title for inclusion. Searches were similarly restricted to the English language. Searches were restricted to [PDF] in Google to limit literature to reports (websites or blogs were not included).

#### Hand searching

Lastly, a reviewer (AJ) hand-searched the reference lists of included reports for relevant literature that may have not been picked up in database searches. Any further identified literature was subject to the same inclusion and exclusion criteria as the literature identified through database searches.

### Selecting studies

One reviewer (AJ) conducted the screening of all 2775 abstracts. Three reviewers (AP, MU, SW) split up the screening to serve as secondary reviewers on at least one-third of the abstracts, and tie-breakers on abstracts they did not screen. This was conducted to limit reviewer bias and ensure the selection criteria were understood and applied the same way by all reviewers. 139 full text reports were subsequently retrieved and screened by all reviewers in a similar manner (AJ, AP, MU, PO, SW). Forty-two abstracts eligible for full-text screening were ultimately excluded because the full literature source could not be located or accessed, even after attempts to contact the authors. The full-text review resulted in 29 studies eligible for final inclusion.

The exclusion criteria were applied sequentially in the following order: (1) if there was no intervention, (2) if the intervention did not target an immigrant population specifically, (3) if the intervention did not have an objective of reducing an adverse health outcome, (4) if the health outcome was not an explicit result of gender bias. Sequential exclusion was chosen on the basis of the large yields of results; it facilitated excluding a large number of ineligible results but also ensured that we would capture every component of our research question.

Articles were included in the analysis if there was a clear description of an intervention that reported the impact on an immigrant population. Therefore, population-wide policies or legislative reforms were excluded, unless there was mention of how the reform affected a specific immigrant subgroup, or immigrants in general (as opposed to the entire population). Furthermore, the intervention’s aim had to focus on reducing health outcomes as result of imported gender bias. For this reason, interventions promoting cervical cancer screening or diabetes prevention were excluded, as those outcomes were more related to physical health-seeking behaviours than to inequitable power relations that shape gender disparities. Several studies were excluded in the final review because they were inaccessible to the reviewers.

### Data management and extraction

EndNote was used to manage retrieved references. Excel was used to create databases of the results for screening.

A data extraction form was compiled with input from all reviewers. The data extraction form was pilot tested on a random sample of 5 articles and revised accordingly. One reviewer (AJ) used this chart to synthesize the results, and extract key information from the 29 included studies.

Relevant characteristics extracted were descriptive characteristics of the intervention, such as its design, location, objective and outcome, and the gender, age group, and country of origin of the participants. We were also interested in whether interventions were evaluated, which helped identify whether the objective was achieved, as well as the source authors’ recommendations for replication. The lessons learned as stated by source authors were also summarized to inform our understanding of characteristics which contributed to the efficacy of the interventions.

## Results

**Fig. 1:**
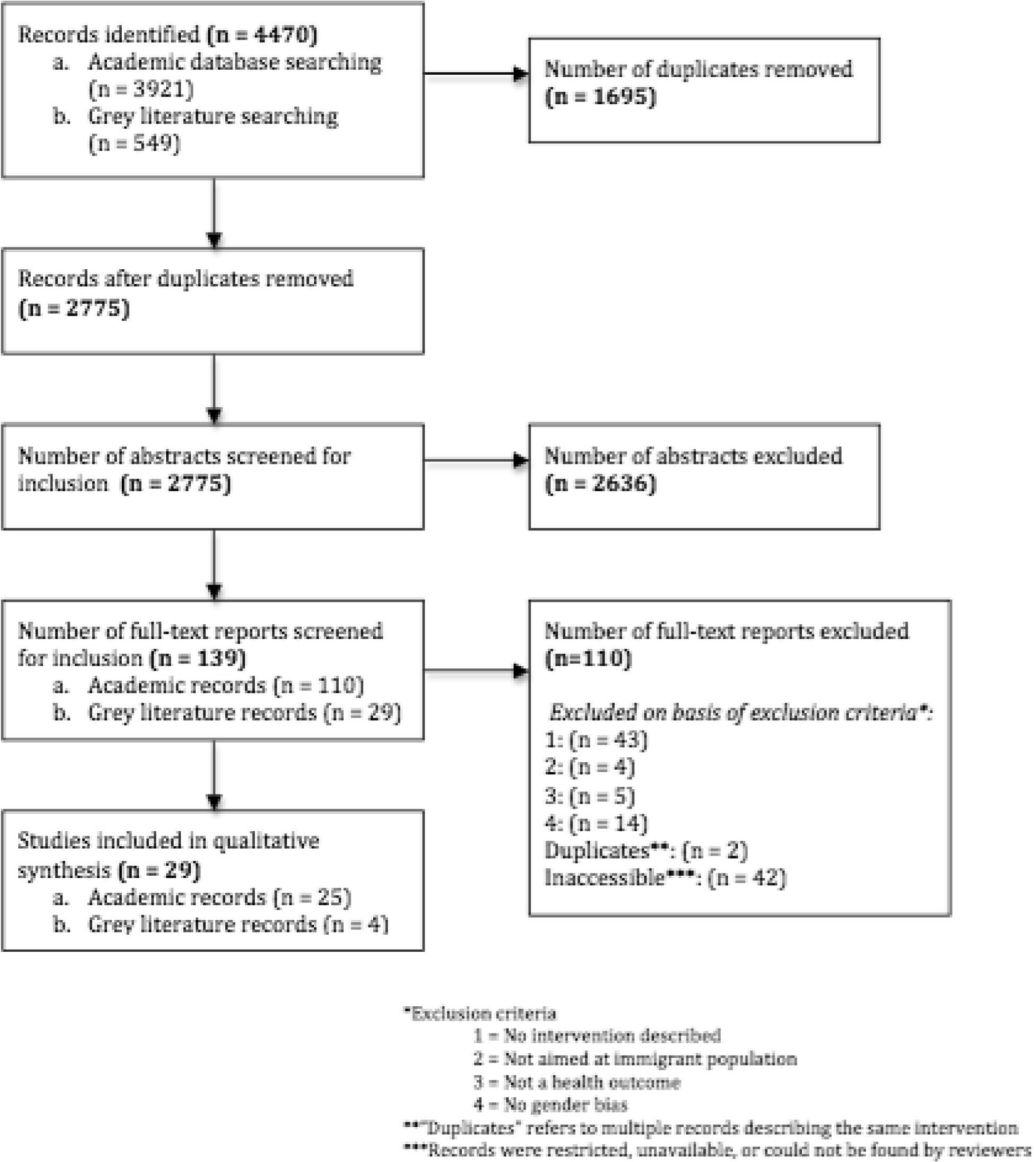
PRISMA Flow Chart of Selected Studies

Twenty-nine studies met the final inclusion criteria, and were included in the analysis. A PRISMA flowchart of the study selection can be seen in Fig. 1.

### Study characteristics

An overview of the main characteristics of the included studies, sorted by outcome of interest, can be seen in Table 1.

**Table 1:** Descriptive Study Characteristics by Outcome of Interest

Gender Outcome	Intervention type	Target population	Gender	Location	Was program assessed? (Yes/No); Approach	Author, year
Domestic violence (n = 17; 58.6%)	Counselling	General	Men	Spain	Yes; Follow up surveys	Echauri et al., 2013
		South Asian	Women	Canada	No	Agnew, 1998
		Chinese	Women	Hong Kong, China	Yes; A questionnaire at 3 time points	Wong et al., 2013
	Educational	African	General	Australia	Yes; Community advisory focus groups	Gregory et al., 2013
		Ethiopian	General	Israel	Yes; Feedback forms, focus groups	Ben-Porat, 2010
		General	Women	Canada	Yes; Focus groups	Heinonen et al., 2006
		Latina	Women	USA	Yes; Focus groups at 2 follow up points	Marrs Fuchsel, 2007
		Latino	Men	USA	Yes; Baseline and follow up questionnaires, in-depth interviews	Celaya-Alston, 2010
		Latino	Men	USA	Yes; Baseline and follow up questionnaires, follow-up interviews with facilitators	Nelson et al., 2010
		Latino	Men	USA	No	Hancock et al., 2009
		Vietnamese, Latino, Somali	General	USA	No	Pan et al., 2006
	Legislative	General	General	USA	No	Adams and Campbell, 2012
		General	Women	USA	No	Orloff and Kelly, 1995
		Latina	Women	USA	Yes; Baseline and follow up surveys	Cesario et al., 2014
	Outreach	Arab	General	USA	Yes; Focus groups	Kulwicki and Miller, 1999
		Arab	General	Canada	No	Baobaid, N.D.
		General	General	New Zealand	No	Robinson and Liu, 2015
Female genital circumcision (n = 2; 6.9%)	Legislative	General	General	Australia	No	Patrick, 2001
		General	General	Italy	No	Turillazzi and Fineschi, 2007
Intimate partner violence (*n* = 6, 20.6%)	Counselling	General	Men	USA	Yes; Intake and discharge surveys	Rothman et al., 2007
	Educational	General	Women	USA	No	Frohmann, 2005
		Latina	Women	USA	Yes; Participant journals and questionnaires at 3 time points.	Serrata, 2012
		Latino	Men	USA	Yes; Interviews with participants	Parra-Cardona et al., 2013
		Latina	Women	USA	Yes; Oral interviews and participant observation of 5 support group sessions.	Morales-Campos et al., 2009
	Outreach	South Asian	General	USA	No	Yoshihama et al., 2012
Sexual violence (n = 4; 13.8%)	Educational	Central and East African	Women	Democratic Republic of Congo, Western Ethiopia.	Yes; Focus groups, interviews, social mapping exercises, attendance records, service provider quality checklists.	Falb et al., 2016
		African	Women	Guinea, West Africa	Yes; Closed-ended interview and written test of literacy skills administered to respondents.	McGinn and Allen, 2006
		African	General	Tanzania	No	UN High Commissioner for Refugees, 1997
	Legislative	Nigerian	General	Italy	No	Baye and Heumann, 2014

#### Outcomes of interest

The outcomes were defined and categorized by reviewers AJ, AP, MU and SW. Of the twenty-nine interventions included, the vast majority targeted domestic violence (referring to violence perpetrated within the home against a person in any family relationship) (*n* = 17; 58.6%). Several of these interventions used the terms “family violence”, particularly among studies where the target population were Muslim or Arab. However, the definition of family violence is captured by our definition of domestic violence. Other interventions also more directly targeted intimate partner violence, which is violence perpetrated by a current or former romantic or sexual partner (*n* = 6, 20.6%). Four interventions were aimed at reducing sexual violence (13.8%), defined broadly as a sexual act committed against someone without freely given consent; this included interventions that were not explicitly aimed at DV or IPV, or referred to violent behaviours at a community or institutional level. Two interventions targeted female genital circumcision (6.9%).

#### Intervention characteristics

More than half of the interventions took place in North America (*n* = 18; 62%), with the majority in the United States of America (*n* = 15; 51.7%), and the rest in Canada (*n* = 3; 10.3%). Of these interventions, all eighteen targeted domestic violence or intimate partner violence. Three interventions took place in African refugee camps (Democratic Republic of Congo, Guinea, Tanzania), with all three targeting the reduction of sexual violence amongst refugee populations (*n* = 3; 10.3%). There were three interventions in both the Pacific region [Australia (*n* = 2), New Zealand] and in Europe [Italy (n = 2), Spain], and one intervention each in China and Israel.

Interventions took many forms. The most frequent type of intervention were educational programs (such as workshops, classes, or curriculums) (*n* = 15; 51.7%). These types of programs varied in terms of the target population and gender, but tended to focus on raising awareness or reducing the prevalence of DV, IPV, and sexual violence. Six of the identified studies discussed legislative reforms (20.6%); most of these reforms were meant to deter a particular practice (FGC), or to facilitate immigration protections for abused women. Four interventions aimed at providing group counseling, either in the form of therapeutic treatment directed at perpetrators of violence or support for survivors (*n* = 4; 13.8%). There were also four outreach interventions in the form of mass media campaigns (*n* = 4; 13.8%), using posters, brochures or television ads in the target population’s native languages.

More than half of the included articles evaluated their respective intervention in some manner (*n* = 16; 55.2%). A variety of approaches were used to evaluate or describe whether the interventions worked or did not work, including focus groups, in-depth interviews, and questionnaires. The majority of approaches were outcome evaluations, aiming to describe how the intervention fared on certain objectives or outcomes after it was implemented, but a few reported an evaluation of the process of implementation. The rest of the interventions mentioned no component of an evaluation (*n* = 13; 44.8%).

#### Study population

Interventions were classified by AJ on the basis of whether they explicitly targeted a subpopulation of immigrants; if none was mentioned, it was assumed to target the “general” immigrant population. Of the twenty-nine interventions included in this study, ten were aimed at a general immigrant population (34.5%). The majority of these were legislative reforms and therefore theoretically population-wide. Eight interventions were explicitly aimed at the Latino population (27.5%); however the majority of these participants were Mexican immigrants in the U.S. Six interventions targeted African communities (20.6%), either in countries of settlement or in refugee camps within Africa. Three interventions were aimed at Asian and South Asian populations (10.3%), and two were aimed at communities of Arabic origin in the United States (6.8%).

A vast number of interventions were generally aimed at an entire community (*n* = 12; 41.4%), regardless of gender. These were typically population-based interventions, such as some legislative reforms or media and outreach campaigns. Of the remaining interventions, eleven exclusively targeted women (38%), while six were directed at men (20.6%). All male-specific interventions worked towards reducing DV or IPV and typically targeted former perpetrators of violence against women.

### Main themes

As per this scoping review’s secondary objective, we were also interested in examining whether the objectives of the interventions were achieved, and if so, what aspects of the intervention the authors attributed their success or failure to, as well as recommendations for future replication. These are summarized in [Additional File 1].

Of the twenty-nine included interventions, twenty-one clearly stated they met their objective (72.4%). However, in a few cases it was unclear whether the objective was met because of the lack of specific reporting or follow-up.

Many of the reports included authors identifying the lessons learned from conducting these interventions; these reflections and recommendations have been amalgamated across the included literature by reviewer AJ, and grouped in the following six major themes:

#### (1) Considering context

Some studies emphasized that context matters; many authors stated the importance of having interventions address the unique cultural, social and institutional determinants in a migrant woman’s life (i.e., feelings of social isolation, tenuous legal status, language barriers, difficulty securing employment). Some studies targeting legislative reforms encountered obstacles to program enrollment and efficiency, by failing to address barriers such as the difficulty obtaining documentation or evidence of abuse [10, 11]. For example, legislative reforms, such as the Violence Against Women Act, require a woman to carefully document abuse and acquire all necessary supporting documentation before submitting a petition; though authors identified that language barriers, limited resources and lack of awareness serve as barriers for immigrant women hoping to benefit from these reforms [10]. Authors recommended that these policies should be supplemented with efforts to increase social, legal, and economic support [12]. Furthermore, since legislative reforms are institutional-level responses, while they can be coordinated nationally, they should be adapted and implemented at the community level, and coordinated with complementary service provision in the community [13]. As much of this literature posits, interventions are most successful when they are multidimensional, and addresses the cultural and socio-economic determinants of health unique to immigrant populations [14].

#### (2) Community engagement

In order to implement anti-oppressive and sustainable interventions, many of the interventions engaged in dialogues with community members which informed intervention design and implementation [15–17]. Some authors found that community leaders (elders, Imams) were more effective in disseminating messages, resolving conflicts, and leading the implementation of various initiatives [18, 19]. Dialogue between community members and intervention facilitators also allowed the implementation of culturally appropriate services that were unique to the needs of those communities [15, 16]. Furthermore, authors found that engaging community members through fostering the ability for open discussion, allowed space for feedback, avoided the use of stigmatizing dialogue and ensured project sustainability [20]. Moreover, engaging with male members as stakeholders was identified by program participants and facilitators as a way to improve lay knowledge and change attitudes [21].

#### (3) Tailoring the approach

Authors stated that interventions were successful when they used approaches tailored to the needs, skills, and abilities of their immigrant population. A few interventions overcame language and literacy barriers, by using self-reflective exercises, group discussion activities, support groups, and arts-based tools [16, 22, 23]. These strategies were identified by authors as fostering behavioural change at the individual and community level, and enabled the sharing of information in a non-traditional educational format [24].

#### (4) Tensions between program delivery and community needs

Two articles identified the challenges posed by prioritizing culturally appropriate and sensitive services to immigrants. One challenge was that prioritizing cultural sensitivity led to the inability to directly question systemic cultural and patriarchal norms shaping gender inequities. Authors suggested that due to this lack of questioning, political and social change was likely to be slow and moderate [25]. A second article compared a set of interventions targeting domestic violence in New Zealand; the population-based intervention was found to moderately change general attitudes by displaying messages about gender inequity, but the culturally-adapted “ethnic” intervention was better at generating trust between the program facilitators and participants where dialogue around domestic violence needed to be sensitive, and was found to promote collective empowerment within the household [26]. The authors indicated a need for rigorous evaluations into whether fundamental approaches drive the differences seen between population-based or culturally-specific approaches, and whether they affect the efficacy of interventions.

#### (5) Building in evaluations

Amongst the 29 articles, 13 interventions did not mention an explicit component of an evaluation, or discuss a specific evaluation approach to measure the impact of the intervention. A few of the included articles identified a need for better testing, measuring, and reporting of the effects of their interventions. Author recommendations included testing the intervention with larger and more diverse samples, to be able to determine effectiveness in various immigrant subgroups [27]. Two studies also made explicit recommendations to amend their measuring tools (such as implementing pre-intervention baseline tests) to accurately determine impact [28, 29].

#### (6) Targeting men

With respect to age and gender, many interventions were population-based, but some specifically targeted a particular age group or gender. Many authors emphasized the importance of including men for gender-based interventions in order to enact behavioural and attitudinal change, and to inform the implementation of these interventions [18, 21]. Of the interventions that specifically targeted men, many of those were aimed at adult male perpetrators of violence [23, 27, 30, 31] (within which participation was enforced by a court of law for males found of perpetrating violence), while most of the interventions involved men in a community-wide response.

## Discussion

Of the 29 reports that met the full-text inclusion criteria, there was little variability amongst the types of intervention designs applied, populations targeted, and health outcomes addressed. The results addressed forms of violence occurring within the home (DV, IPV), and were largely unique to the Latino immigrant context in the United States of America; few interventions targeted other populations, despite there being a variety of health outcomes and immigrant groups in many other countries of settlement around the world. This indicates opportunities for greater innovation in the design and delivery of gender-based health interventions. However, there were common findings and lessons learned from these interventions; these included the importance of considering the unique contexts of immigrants, engaging with community members, especially young males, and tailoring interventions according to their lived experiences, needs, and abilities. Furthermore, while most of these authors stated to have achieved the objective of their intervention, there remains a need for stronger reporting and evaluation.

### Knowledge Gaps & Directions for research

There are several knowledge gaps identified in this scoping study that suggest particular directions for future research and action.

#### Gap 1: Need for broader representation in the literature

The majority of the literature identified in our review was aimed at reducing domestic violence amongst Latino populations in the USA. This is not surprising, given the significant population of Latino immigrants in the USA. However, this indicates a need for representation of other locations, populations and outcomes in the literature. This is reflected in geography, with a need for more English publications from countries of high immigrant settlement, such as the United Kingdom, Canada, and Australia. For example, despite the foreign-born population accounting for 20.6 and 28.2% of Canada and Australia’s total country population respectively [5, 32], there were very few interventions that took place in either country, indicating a gap of interventions being conducted or published. Furthermore, immigrant populations other than the Latino population are underrepresented in the literature. Many of these interventions were conducted in Spanish or tailored to the Latino context. Due to cultural dissimilarity, interventions applied to Latino populations may not be applicable to other immigrant populations. More research is needed into how these interventions can be adapted and applied to other populations. Moreover, the cultural and traditional beliefs, attitudes, and customs that underpin these patterns of gender bias are unique to particular immigrant groups and need to be understood to effectively design and implement an intervention. Additionally, the lack of representation of several outcomes in the intervention literature suggests a paucity of interventions aimed at other gender-based inequities, such as FGC or prenatal sex selection, despite recent research that these outcomes occur amongst immigrant populations [33, 34]. The majority of interventions were aimed at reducing domestic and intimate partner violence, and though these outcomes are vital to address, it is equally pressing to ensure the publication of interventions targeted at a range of outcomes.

#### Gap 2: Need to include men

The range of intervention approaches targeting men was limited, with the majority of these interventions intended to police behaviours of violent adult male perpetrators. This indicates the need for further research into the mechanisms of targeting adolescent males before patterns of violent behaviour are exhibited. This also indicates that male community members should serve as a significant resource for service providers to engage with, either as participants or to inform the intervention. The need to involve men in these types of interventions is a well-accepted notion; addressing masculine ideals can help form more equitable relationships within the community, promote community change, and address oppression [35]. Furthermore, behaviour change related to violence can be difficult in communities that have experienced trauma or conflict, such as amongst many immigrants or newcomers [35], reinforcing the need for tailored approaches that address the norms and beliefs associated with masculinity in these communities. Lessons learned from humanitarian settings about male engagement can be applied in the migrant context [36]; since typical community and family structures are disrupted in both humanitarian and migrant settings, this creates an opportunity to involve men as a key strategy to navigate a new family dynamic and break down harmful gendered norms.

#### Gap 3: Need for more reflexive reporting by service providers

In order to support evidence-informed programming, more research, evaluation, and knowledge exchange around a diverse number of health outcomes and contexts (including location and target populations of interventions) is needed. Academics, program planners, and service providers are encouraged to share their research, practices, and experiences to help inform interventions for other immigrant populations (with unique contexts depending on countries of origin and settlement) as well as for outcomes that may often be overlooked.

#### Gap 4: Need to address inequitable patriarchal norms

It is difficult to determine how many of these interventions addressed the underlying cultural and patriarchal norms that shape the experiences of gender bias. Most of the interventions achieved their goal of empowerment, behavior change or increasing self-esteem or awareness. However, it is not clear if these results were due to explicit attempts by the facilitator to dismantle inequitable gender norms, or as a by-product of increased knowledge, resources and support. However, as previously discussed, it is challenging for community-based agencies to deconstruct patriarchal gender norms that disadvantage women, while using sensitive approaches that do not stigmatize or marginalize people further. Agencies often face internal tensions between providing appropriate yet sensitive care to their community members, but wanting to target these norms [25]. More research is needed to further examine these tensions and how they may inhibit or facilitate an intervention’s success.

### Implications for future practice

As migration continues to shape countries of resettlement around the globe, health and social service providers in countries of settlement will be faced with responding to the needs to immigrants. Some of these needs may be due to manifestations of gender bias that uniquely disadvantages immigrant women’s health. There will be a need for service providers to respond adequately to this significant population, particularly by implementing evidence-based programs and policies. This also calls for greater institutional and financial support for the spread and scalability of interventions that support this growing population.

The main findings and recommendations identified by the authors contribute to understanding how interventions targeting health outcomes resulting from gender-bias among immigrants should be guided. This is particularly useful for providers in other Western countries who want to replicate these programs. Whilst there is a clear need for further research, there are many recommendations for service providers to be aware of in order to create beneficial and effective interventions. It is evident from the literature that using multidimensional and multi-systemic interventions tailored to the unique context and needs of a specific immigrant population is an effective approach. This is accomplished by engaging with target communities to foster trust and acceptance, and may be enhanced by engaging with male members early on in the process. However, service providers are often limited in their capacity to scale up an intervention in order to respond to a wide range of immigrants’ needs. Instead, they are encouraged to collaborate, coordinate, and integrate their approaches with other service providers to ensure that all these needs can be addressed. These recommendations have been captured in similar reviews of gender-based violence interventions in humanitarian settings [36]; service providers in countries of settlement can apply the lessons learned from these resource-constrained environments.

#### Strengths and limitations

To our knowledge, this scoping review is the first to examine interventions to reduce health outcomes as a result of gender bias against immigrant women. Although this review focused on how gender bias within immigrant communities affected *women* adversely, we recognize that these gender biases could also potentially adversely affect people with non-binary gender identities.

The methodological strengths of this review are the broad search strategy to ensure wide coverage, the use of a variety of academic databases to identify multidisciplinary literature, the use of grey literature searches to capture reports that are not peer-reviewed (and therefore could have been published on the website of an agency), and the use of multiple reviewers for screening.

The literature yielded by our search strategy may be limited for a few reasons. First, we applied a very rigid strategy for the grey literature searches by limiting eligible terms for study inclusion. Therefore, it is possible we missed literature sources that were labelled by different terms in the title. However, when we used a greater number of terms, the search results grew to an unreasonable volume. After initial scans of the titles, we found that these results were irrelevant to our research question and would be excluded based on our criteria. Second, consultations with expert authors and searching key organizations for reports were not undertaken due to time constraints and the high volume of key organizations globally; however hand searching of included articles was conducted to compensate for this. Third, our results were limited to publications in English, as the reviewers only spoke English, which likely excluded reports from important European countries of settlement. There may be a need for further systematic searching of interventions and evaluations in other languages, particularly in regions not captured by this search strategy. Fourth, interventions targeting other social determinants of health that may contribute to mitigating the effects of gender bias (language classes, employment programs, skill training) were not part of our search strategy, though these determinants may have an indirect effect on gender-based health outcomes for immigrant women. An additional limitation is that three included studies took place in refugee camps and not in a traditional country of settlement. There are important contextual differences which would influence the delivery and effectiveness of these interventions in other settings.

Lastly, this scoping review aimed to answer the research question based on the currently available literature. We are unable to accurately discern whether the results are reflective of current immigration trends and the current prevalence of these health outcomes, or if these results are driven by greater awareness of and greater research funding for outcomes such as DV. There are likely many interventions that are not published in the peer-review or grey literature, perhaps because of resource constraints such as the scarcity of funding, interest, or personnel to conduct research or evaluations. If so, our results may not be representative of all interventions actually being conducted.

## Conclusion

There was a dearth of knowledge around interventions being conducted to address manifestations of gender bias in immigrant women’s health in Western countries of settlement. This scoping review intended to fill that gap by mapping out what interventions have been done, identifying areas for future research, and by synthesizing key findings to inform future practice. The lessons learned from these interventions included tailoring the approach to the unique context of immigrants’ lived experiences, and engaging with community members to inform the intervention design and implementation, particularly male members. While there are many recognized efforts in this field, there is a need for the scope of the literature to be broader, and to reflect a growing and changing immigrant population. Furthermore, program planners, service providers, and policy makers are strongly encouraged to share their research, practices and experiences to inform interventions for immigrant populations or health outcomes that were under-represented in the results of this scoping review. It is our hope that the literature identified in this review will be effective in informing future intervention design and implementation to mitigate the health effects of gender bias against immigrant women in many countries of settlement.

## Additional file

Additional file 1: Table 2. Summary of Authors’ Main Findings and Recommendations. (DOCX 27 kb)

## Abbreviations

DV: Domestic Violence
FGC: Female Genital Circumcision
GII: Gender Inequality Index
IPV: Intimate Partner Violence
