# Supplementary material for: Interventions to reduce adverse health outcomes resulting from manifestations of gender bias amongst immigrant populations: a scoping review
Source: BMC Womens Health. 2018 Jun 19;18:104. doi: 10.1186/s12905-018-0604-2 (PMC6008916; doi:10.1186/s12905-018-0604-2)
Supplement: Supplementary file 1 — Table 2. Summary of Authors’ Main Findings and Recommendations. (DOCX 27 kb) [file 12905_2018_604_MOESM1_ESM.docx]

Table 2: Summary of Authors’ Main Findings and Recommendations

| **Outcome of interest** | **Author, year; setting; population** | **Intervention type; Objective** | **Objective achieved; Authors’ main findings** | **Authors’ recommendations for future practice** |
| --- | --- | --- | --- | --- |
| Domestic violence | Adams and Campbell, 2012; USA; all immigrants | Legislative; The Violence Against Women Act (VAWA) allows immigrant women survivors of DV and their children to obtain permanent residence without cooperation of documented spouse or parent. | Partially; VAWA helped abused immigrant women seek citizenship and safety but several barriers remained, including lack of awareness about the reform and IPV, challenges with obtaining evidence of abuse, and difficulty accessing services. | A need for patient education, assistance with documentation, advocacy, provision of unique services for immigrant survivors, and further research into immigrant health needs. |
| Domestic violence | Agnew, 1998; Canada; South Asian women | Counselling; Community-based support and counselling services to reduce incidence of violence in South Asian families by providing culturally appropriate services, and develop public education strategies for South Asians. | Partially; By prioritizing culturally appropriate service provision due to funding constraints, community-based agencies rarely question systemic patriarchal norms that shape gender inequities, in order to be sensitive to clients. Therefore, political and social change is slow and moderate in reducing violence against women. | Community-based agencies need to create spaces for women to articulate their experiences, and encourage them to challenge patriarchal cultural practices and become more political to overcome women's oppression. |
| Domestic violence | Ben-Porat, 2010; Israel; Ethiopian immigrants | Educational; A training program providing Ethiopian social workers with the knowledge, skills and tools to work within their community in culturally sensitive ways to reduce rates of DV. | Yes; Using Ethiopian social workers helped promote empowerment amongst community members. Participants also reported having an enhanced ability to work on this issue within their communities, but also highlighted the importance of focusing on men as an intervention target. | Interventions should address cultural and socio-economic determinants, and should be multi-dimensional and multi-systemic by encouraging collaboration among all parties. |
| Domestic violence | Heinonen et. al, 2006; Canada; all immigrants | Educational; A participatory group workshop for immigrants to discuss DV prevention strategies, and enhance women's capacity to become informal resources in their communities. | Yes; Participants were very satisfied, but indicated a need for more sustained training, as well as resource materials in different languages. Participants noted the importance of engaging immigrant women from the community, but recommended involving men. | "Train the trainer" programs should engage both men and women to improve their lay knowledge and offer sustained training. |
| Domestic violence | Cesario et. al, 2014; USA; Latina women | Legislative; To examine the impact of shelter intervention and protective orders on maternal mental health, resiliency, and further abuse in documented and undocumented immigrant women. | Yes; Program was found to be effective in improving mental health and resilience outcomes, and reducing abuse severity after receiving assistance, irrespective of a woman's documentation status. | Contact with service providers, offering safe shelter and justice services can dramatically improve safety and well-being of abused immigrant women, but efforts should also be made to increase social support, and should consider her immigration status, economic situation and related social deficits. |
| Domestic violence | Echauri et. al, 2013; Spain; all immigrant men | Counselling; A therapeutic treatment program for male perpetrators of DV to prevent relapse by teaching preventative strategies, treating psychopathological symptoms, and enhancing motivational behaviour. | Yes; The program was found to have a very high success rate (87%) and shown to be effective in reducing violent behaviours. Similar levels of effectiveness were found amongst citizen and immigrant perpetrators. | Could be used successfully with immigrant populations, but should be tested with larger and more diverse samples. |
| Domestic violence | Gregory et. al, 2013; Australia; African immigrants | Educational; STAMP training curriculum is delivered to community leaders to promote nonviolent conflict resolution, and to develop formal communication channels between community leaders and justice system. | Yes; The intervention brought about a positive attitudinal change about gender roles and an increased knowledge of the Australian legal system amongst community leaders who participated. Community leaders and service providers also developed stronger relationships. | Engaging men and women as facilitators can promote reflective group discussion sessions.Engaging community leaders can strengthen appropriate delivery of services. |
| Domestic violence | Kulwicki and Miller, 1999; USA; Arab immigrants | Outreach; To develop an outreach campaign to educate community members on DV, and to implement a mass media campaign providing relevant Arabic materials. | Yes; As a result of the media campaign, community members indicated an increased awareness of DV risk factors and community resources available to them. | Dialogues with community members found a need for culturally and linguistically sensitive educational programs tailored for low-income and illiterate members, that also target the environmental conditions that influence DV. |
| Domestic violence | Marrs Fuchsel, 2007; USA; Latina women | Educational; Si Yo Puedo, weekly educational workshops that provide participants with education about DV and self-esteem | Yes; Participants described improvement in self esteem and knowledge of DV prevention, as well as a decrease in violent behaviours in relationships. | Valuable culturally relevant curriculum, especially among ILW. Use of follow-up groups helps expose ILW to other programs and services they may need. |
| Domestic violence | Nelson et. al, 2010; USA; Latino men | Educational; To decrease IPV through a weekly series of reflective exercises and discussion groups meant to promote behavioural change. | Yes; Group discussion formats allowed participants to self-reflect at their own pace, and fostered knowledge exchanges between facilitators and participants. | Interventions based in self-reflection discussion groups can be successful in promoting individual and community behaviour change around violent behaviour. |
| Domestic violence | Orloff and Kelly, 1995; USA; all immigrant women | Legislative; To implement specific guideline into VAWA to use when evaluating gender-related asylum claims brought forth by women. | Unclear; This reform advances legal protection for abused migrant women by incorporating gender-specific guidelines. At time of publication, only Canada and USA were using the guidelines. | Further research is needed into how this reform can evolve. Advocates should share strategies to build a better immigration system for abused women. |
| Domestic violence | Pan et. al, 2006; USA; Vietnamese, Latino, Somali immigrants | Educational; A collaborative project between community and service providers to increase awareness of DV and develop culturally specific programs for each community. | Yes; Engaging with different immigrant communities developed the opportunity to focus on family harmony as the context within which to address DV. Hiring and training bilingual staff contributed towards building community relationships that informed intervention design. | Cultural values and beliefs are specific to each immigrant community, and must be identified through community engagement in order to develop effective interventions. |
| Domestic violence | Robinson and Liu, 2015; New Zealand; all immigrants | Outreach; Comparing two interventions that target DV: (1) A mass media outreach campaign to change community-level attitudes,and raise awareness; (2) to provide targeted social services and programming to migrant and refugee-background communities. | Yes; Both interventions were found to promote empowerment. The population-based intervention aimed to change attitudes by targeting gender inequities. However, the ethnic intervention promoted collective empowerment within the household, and was better at generating trust in communities where dialogue around domestic violence needs to be sensitive. | Approaches can be used to supplement each other, but there is a need for rigorous evaluation into whether ideological beliefs drive differences between mainstream or ethnic interventions. |
| Domestic violence | Hancock et. al, 2009; USA; Latino men | Educational; Group discussion sessions to teach male perpetrators strategies for healing and curbing aggressive behaviour in order to reduce DV. | Yes; Participants noted having changed beliefs about traditional gender roles and oppression, which was enhanced by having a female facilitator who could provide a different perspective to discussions. | When constructing culturally sensitive approaches, consider legal status, length of time in receiving country, and contexts of countries of origin and settlement. |
| Domestic violence | Baobaid, N.D.; Canada; Arab immigrants | Outreach; Community engagement project to promote dialogue between the Muslim community and mainstream anti-violence agencies, and to enable the mobilization of the Muslim community on DV. | Yes; Strong involvement of Muslim community and service providers resulted in better capacity building (examples include incorporating Canadian law and Islamic teachings, including Muslim men in project activities, engaging with religious leaders). | It's important to foster open discussion spaces and avoid stigmatizing dialogue when engaging with community members for capacity building. |
| Domestic violence | Celaya-Alston, 2010; USA; Latino men | Educational; To develop a Spanish-language curriculum to address DV with immigrant Mexican men. | Yes; The discussion group was found to promote knowledge sharing between participants and resulted in an improvement of DV knowledge. | The program was successful because of it's male-focused approach, engagement with community advisors, use of diverse teaching methods adapted for low literacy populations, and emphasis on primary prevention. |
| Domestic violence | Wong et. al, 2013; Hong Kong; Mainland Chinese women | Counselling; To counsel, empower, and support abused women, and to help create a specific safety plan for each individual. | No; The intervention was effective at reducing depressive symptoms in abused immigrant women for a short-term period, but this was not sustained. There was no indication of changes in DV prevalence. | Attention should be focused on strengthening capacity of immigrant women, by providing tips for typical DV challenges, knowledge of the legal system, and making referrals to community resources. |
| Female genital circumcision | Patrick, 2001; Australia; all immigrants | Legislative; To review national and provincial Australian policies that criminalize aiding or abetting the practice of FGC | Partially; Legal measures should be seen as a last resort, and should be coupled with community education initiatives that target wider socioeconomic determinants of migration in order to minimize harm. | FGC policies are most successful if they are coordinated nationally but adapted locally, coordinated with other forms of service provision, work with communities to create culturally inclusive responses, and avoid excessive coercive strategies. |
| Female genital circumcision | Turillazzi and Fineschi, 2007; Italy; all immigrants | Legislative; Parliamentary law that lays down the necessary measures to prevent, contrast and suppress FGC practices as violations of the individual's fundamental rights to physical and mental integrity. | Unclear; The regulation was demanded by activists, social workers in affected communities, and parliamentarians, and applauded by Amnesty International; however little indication about effectiveness of policy on deterring FGC. | Legislative reforms must retain an explicit reference regarding the difficulties proving a woman's consent to FGC. Legislation was supplemented with financial investment into prevention programs. |
| Intimate partner violence | Frohmann, 2005; USA; Latina & South Asian women | Educational; The Framing Safety project provides survivors of IPV with an arts-based tool to explore their lived experiences with abuse, and to educate communities about violence prevalence and prevention. | Yes; Project goals of empowerment and community education were met. | Arts-based approach gave participants a voice and a tool to capture complex circumstances, but it is a difficult and expensive approach. |
| Intimate partner violence | Morales-Campos et. al, 2009; US; Latina women | Educational; A support group for women currently in an abusive relationship to develop strategies and a safety plan, and learn about resources. | Yes; The support group was found to have fostered a sense of community amongst members, who shared coping tools and strategies. | Culturally appropriate support group interventions can have a positive effect for women living in situations of violence. |
| Intimate partner violence | Parra-Cardona et. al, 2013; USA; Latino men | Educational; Workshops to promote behavioural change amongst males through an understanding of patriarchy, to learn interpersonal skills to support that behaviour change, and to teach violence prevention strategies. | Yes; Participants expressed changes in attitudes, and an awareness and active use of violence prevention strategies (such as a time out). | The Duluth intervention is beneficial for immigrants if implemented in a culturally relevant way, but should provide more diverse services over a longer period. |
| Intimate partner violence | Rothman et. al, 2007; USA; all immigrant men | Counselling; Court-mandated counselling for perpetrators that enable them to develop new non abusive attitudes and behaviours. | Yes; Immigrants were found to be more likely to complete the program, but due to fears of deportation as a result of violating probation. No differences were found between non-English and English speakers, indicating potential generalizability to immigrant populations. | More research is needed into whether culturally specific programs offer improvements over mainstream programming. |
| Intimate partner violence | Yoshihama et. al, 2012; USA; South Asian immigrants | Outreach; A mass media campaign to create new community norms that denounce IPV and encourage bystanders to confront abusive behaviour | Yes; Meaningful participation from community members in this project helped build community capacity. The program successfully targeted institutional and societal factors, while adapting to the local community. | While it is challenging to translate broad behaviour change interventions to target a specific immigrant population, it is critical to engage with members at the individual, social network, and community level in order to ensure project sustainability. |
| Intimate partner violence | Serrata, 2012; US; Latina women | Educational; A training program to promote leadership development, self-empowerment, and outreach skills as a way to develop existing strengths among Latina survivors of IPV. | Yes; The intervention was effective in improving self-empowerment as a result of enhanced leadership skills, but IPV knowledge levels were not influenced by the intervention (though baseline levels were very high to begin with). | Use different measures to evaluate the IPV component accordingly, or use existing measures with participants who have no prior experience with an IPV intervention. |
| Sexual violence | Baye and Heumann, 2014; Italy; Nigerian women | Legislative; The Social Protection Program assists debt-bonded migrant sex workers experiencing SV by providing health and legal information, securing legal documents, facilitating shelter stays. | No; Program found to have barriers that make enrollment challenging, and fails to address the vulnerabilities faced by migrant sex workers. | Programs should be collaborative among stakeholders and should address the legal and social complexity of undocumented migrant sex workers. |
| Sexual violence | Falb et. al, 2016; Dem. Republic of Congo, Ethiopia; Central and East African women | Educational; COMPASS program provides opportunities for girls to build assets to protect against violence and through mentorship, learning, and peer interaction in safe spaces. | N/A; No key findings because study is ongoing. | Rigorous impact research is needed in humanitarian emergencies, particularly through strong engagement with local communities and team members. |
| Sexual violence | McGinn and Allen, 2006; Guinea, West Africa; African women | Educational; An adult literacy program to teach literacy skills, and gender-based violence awareness, and increase usage of reproductive health services in refugee camps. | Yes; Participants were found to have a high retention of material taught in reproductive literacy classes and higher self esteem, but there were no explicit findings on gender-based violence reduction. | Worth replicating in other refugee camps, with pre-intervention baseline measures implemented to accurately measure impact. |
| Sexual violence | United Nations High Commissioner for Refugees, 1997; Tanzania; African immigrants | Educational; A program to train community members to offer support and act as a liaison between sexual assault survivors and service providers, and to lower the incidence of sexual violence in camps through an awareness campaign. | Yes; The awareness campaign brought about a greater knowledge of sexual violence prevention, which resulted in higher rates of reporting of assaults and improved medical attention. | Trained CIT members were better able at engaging community members, supporting victims, and reducing risk factors within the camp. There was a need for initial cross-sectoral collaboration and frequent refresher trainings. |
